# A portable fiber-optic raman spectrometer concept for evaluation of mineral content within enamel tissue

**DOI:** 10.4317/jced.53185

**Published:** 2017-02-01

**Authors:** Anna Akkus, Shan Yang, Renato Roperto, Hathem Mustafa, Sorin Teich, Ozan Akkus

**Affiliations:** 1 PhD, CWRU School of Dental Medicine, 2124 Cornell Road, Cleveland, OH 44106-4905; 2 PhD, Jackson State University, Department of Physics, Atmospheric Science and Geophysics, 1400 John R. Lynch St Jackson, MS 39217; 3 DDS, MSc, PhD, CWRU School of Dental Medicine, 2124 Cornell Road, Cleveland, OH 44106-4905; 4 PhD, Department of Mechanical and Aerospace Engineering, Case Western Reserve University, 10900 Euclid Ave.Cleveland, Ohio 44106-7222; 5 DDS, MBA, CWRU School of Dental Medicine, 2124 Cornell Road, Cleveland, OH 44106-4905

## Abstract

**Background:**

Measurement of tooth enamel mineralization using a clinically viable method is essential since variation of mineralization may be used to monitor caries risk or in assessing the effectiveness of remineralization therapy. Fiber optic Raman systems are becoming more affordable and popular in context of biomedical applications. However, the applicability of fiber optic Raman systems for measurement of mineral content within enamel tissue has not been elucidated significantly in the prior literature.

**Material and Methods:**

Human teeth with varying degrees of enamel mineralization were selected. In addition alligator, boar and buffalo teeth which have increasing amount of mineral content, respectively, were also included as another set of samples. Reference Raman measurements of mineralization were performed using a high-fidelity confocal Raman microscope.

**Results:**

Analysis of human teeth by research grade Raman system indicated a 2-fold difference in the Raman intensities of v1 symmetric-stretch bands of mineral-related phosphate bonds and 7-fold increase in mineral related Raman intensities of animal teeth. However, fiber optic system failed to resolve the differences in the mineralization of human teeth.

**Conclusions:**

These results indicate that the sampling volume of fiber optic systems extends to the underlying dentin and that confocal aperture modification is essential to limit the sampling volume to within the enamel. Further research efforts will focus on putting together portable Raman systems integrated with confocal fiber probe.

** Key words:**Enamel, mineral content, raman spectroscopy.

## Introduction

Dental enamel is 95% mineral and 1% organic component and 4-5% water by weight percentage (1-5). A reduction in mineral content has direct consequences as far as dental ailments are concerned. For example, molar-incisor hypomineralization increases tooth sensitivity to food, drinks, and thermal changes as well as results in restoration failure (6). Moreover, previous studies suggest a possible relationship between enamel mineral concentration and caries susceptibility (7-9).

Several authors have investigated enamel mineral content (10-14) using various characterization methods in the context of decay (8,9), and demineralization/ remineralization processes (15,16), age (17,18) and disease (19,20). Micro-computed tomography (micro-CT) was used to investigate mineral densities as well as elemental content in different layers of healthy human enamel based on the age of the individual (10,17). However, such radiographic methods are limited to *ex vivo* laboratory conditions and destructive to the specimens.

An understanding of tooth enamel mineralization using a clinically viable method is essential since variations in mineralization may serve as an early predictor of a dental health, and may indicate high populational susceptibility to caries. Raman spectroscopy is one of the few methods which offers the opportunity to study enamel mineralization non-destructively *in vivo* (21,22). While Raman spectroscopy has been used to assess tooth mineralization, there are no studies that examined mineralization of the enamel systematically vis a vis enamel mineralization of various animals as well as differentiation between of high mineralized and low mineralized enamel of different individuals. The current study employed a portable fiber optic Raman probe to identify the variations of mineralization of enamel between individuals and animal controls with high and low mineralization of the enamel. Results of the fiber optic Raman system was compared to the results obtained by using a research-grade commercial Raman microscope. The study also developed a confocal fiber optic set up to confine the sampling volume to the enamel specifically and to eliminate interference from the dentin.

## Material and Methods

-Sample Preparation

**Figure 1:**
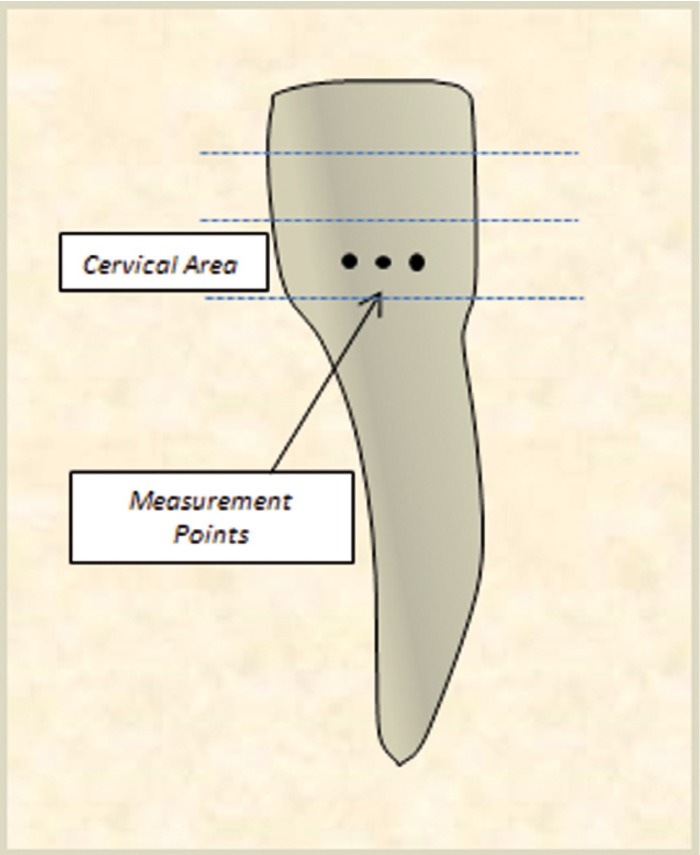
Schematic of the tooth examination sites with various Raman techniques.

Human teeth were obtained in compliance with the National Institute of Health guidelines. The Institutional Review Board exemption was filed and approved (Protocol#: EM-13-17). Adult human incisors were extracted as a part of a normal treatment plan. The teeth were collected fresh within the date of the extraction and kept moist at all times without any additional disinfecting treatment. A dentist assessed the enamel of the specimens selected for Raman analysis in order to ensure that healthy intact enamel was evaluated. The samples were wrapped in wet tissue paper individually and stored in a -20ºC freezer. Prior to Raman analysis the specimens were thawed at room temperature for 30 minutes while being wrapped in moist tissue paper. The crown of the tooth was measured with a ruler and the lower third of the tooth, cervical area was examined with various Raman spectrometers (Fig. 1).

Animal teeth from alligator, boar and buffalo with various levels of enamel mineralization were also included as a phantom sample set. There is a considerable variation in mineralization of calcified tissues (23,24) where the ash masses are ranging from 45% to more than 90%.

-Raman Spectroscopy

A high-fidelity confocal Raman microscope (Xplora, Horiba Jobin Yvon, NJ) was used to investigate the enamel mineralization. Measurements of this research-grade system constituted as a reference point to which the fiber optic systems’ measurements were compared to. The Raman microscope is composed of a laser source at 785 nm, and measurements were performed using a 1200 lines/mm grating, which provided a spectral dispersion of 0.8 cm-1 /pixel leading to 4 cm-1 spectral resolution. Labspec software was used for both acquiring the data and for performing background subtraction from the spectra.

The fiber optic Raman set up is constructed based on a 785 nm fiber laser (Innovative Photonic Solutions) with 100 mW output power, a Raman spectrometer and a fiber probe combination (Wasatch Photonics, NC) as illustrated in figure 1. The thermo-cooled Raman spectrometer contained a high sensitive back-thinned low etaloning CCD sensor (Hamamatsu S10420-1006, NJ) and a gelatin-based volume phase holographic transmission grating (Wasatch Photonics, NC) which enabled high optical throughput. The spectral resolution was 10 cm-1 with a spectral dispersion of 1.7 cm-1/pixel at 50 µm slit width. The laser light is transmitted through an optical fiber to the Raman probe which delivered the laser to the sample; the same fiber probe also collected the Raman signals from the sample and sent to the spectrometer through another fiber.

## Results

**Figure 2:**
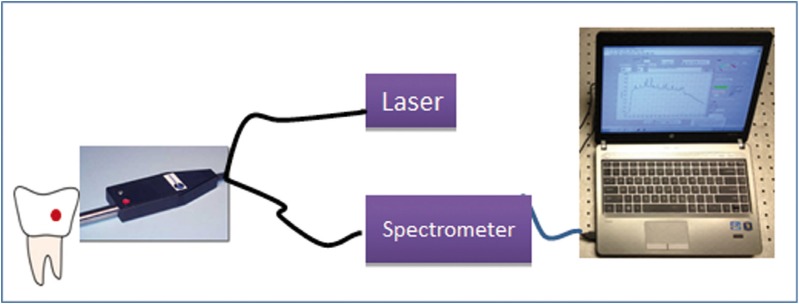
Custom designed fiber-optic Raman system.

**Figure 3:**
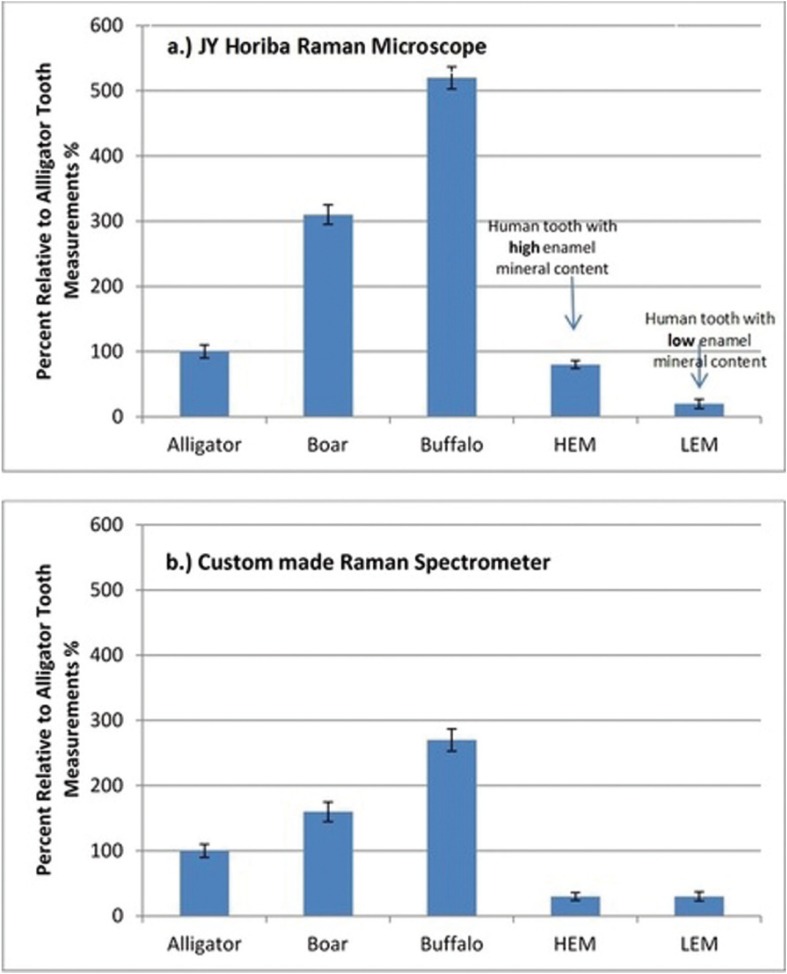
a) Enamel mineralization of various animal teeth accessed with commercially available JY Horiba microscope. b) Enamel mineralization of various animal teeth accessed with and custom made, fiber optic Raman set-up.

Enamel mineralization levels have varied substantially between animal teeth (Fig. 2) when it was evaluated with the research grade Horiba Jobin-Yvon confocal microscope. The highest Raman-based mineralization intensity was observed for the buffalo tooth which had 5-fold greater mineralization than the alligator tooth. Boar tusk had an intermediate level of mineralization. A similar trend of mineralization between animal teeth was also observed when the mineralization scores for the animal teeth were assessed with the non-confocal custom-made set up (Fig. 3). The fold increase in mineralization between alligator and buffalo enamel as observed by the fiber optic set-up was 2.5 fold.

Illustrates the mineralization score measurements obtained by using research-grade Raman microscope, custom-made fiber optic set up as well as modified confocal fiber optic set-up for two human teeth with varying mineralization levels. Research-grade system demonstrated that one tooth had higher enamel mineralization (HEM) than the other tooth. low enamel mineralization (LEM) scores. The set up shown in figure 1 was not able to differentiate between the HEM and LEM teeth, however JY Horiba system allowed to identify high and low mineralized human teeth.

## Discussion

We used research grade confocal system as the reference for mineralization measurement. Raman has been standard in the literature of mineralized tissues for mineralization assessment (22,24,25). Future studies will require formal measurements of mineralization, such as calcium content by back scattered electron microscopy or ash content analysis to calibrate the Raman intensities of the confocal fiber optic system with direct measurements of mineralization. Future studies are also needed to quantify the depth of penetration of the laser by varying confocality.

It has been suggested that the lower mineral concentration may be translated into increased porosity and is possibly linked to higher caries susceptibility (8,9). In addition, some studies (7,8) hypothesized that mineral concentration may be a factor determining rate of demineralization/remineralization as well. Epidemiological studies demonstrate that children from lower social background have higher caries rate (9). It is unknown whether enamel mineralization also plays a role in the greater caries-risk in this population. Other effectors of enamel mineralization are poor oral hygiene, alcohol consumption and high intake of dietary carbohydrates. An early identification of the individual with overall low mineralization of the enamel may be a valuable screening tool in determining a group with much higher than average caries risk, allowing intervention before development of caries.
